# Supplementary material for: Comparing a computational model of visual problem solving with human vision on a difficult vision task
Source: PLoS Comput Biol. 2025 Dec 9;21(12):e1012968. doi: 10.1371/journal.pcbi.1012968 (PMC12707649; doi:10.1371/journal.pcbi.1012968)
Supplement: S5 Text — (PDF) [file pcbi.1012968.s005.pdf]

S5 Text. Examples of images generated by GANs Sample images from the GAN models trained on MNIST [1] and Fashion MNIST [2] dataset are given in Fig 1

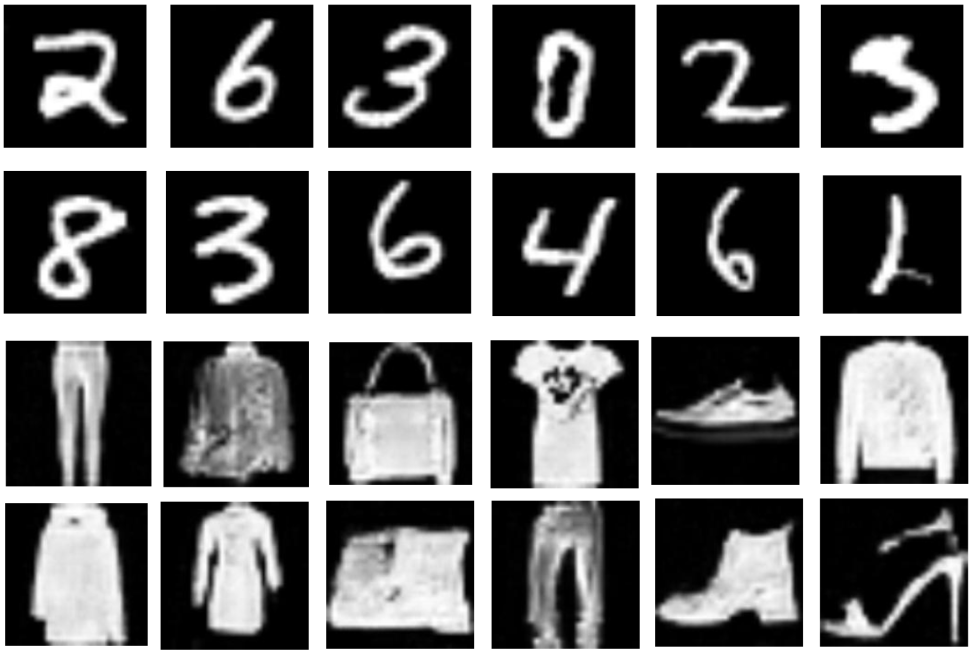

Fig 1. Examples of images generated by the GANs The figure shows images sampled from the GANs used in the GenSearch algorithm. Each image in this panel is generated using the GAN models that we trained on the popular datasets MNIST and Fashion MNIST.

References

1. LeCun Y, Bottou L, Bengio Y, Haffner P. Gradient-based learning applied to document recognition. Proceedings of the IEEE. 1998;86(11):2278–2324.

2. Xiao H, Rasul K, Vollgraf R. Fashion-mnist: a novel image dataset for benchmarking machine learning algorithms. arXiv preprint arXiv:170807747. 2017;.
