# Supplementary material for: Comparing a computational model of visual problem solving with human vision on a difficult vision task
Source: PLoS Comput Biol. 2025 Dec 9;21(12):e1012968. doi: 10.1371/journal.pcbi.1012968 (PMC12707649; doi:10.1371/journal.pcbi.1012968)
Supplement: S6 Text — (PDF) [file pcbi.1012968.s006.pdf]

S6 Text. Analysis of GenSearch with lower population size We restrict the width of the population and the number of parents to 100 and 20 respectively while increasing the maximum generations allowed by the same factor to 300. We then did a hyperparameter search for best crossover and mutation rates for this setup. With the mutation rate of 0.2 and a crossover rate of 0.01. The best resulting setup still has a lower accuracy of 0.44 than the original accuracy of 0.66 for MNIST dataset. We understand that the lower accuracy in this setting is expected as by their nature evolutionary algorithms with lower population have the tendency to have a lower genetic diversity and can converge prematurely to a more local solution. The population size in that manner represents the exploration vs compute trade off in genetic search.
