# Supplementary material for: Comparing a computational model of visual problem solving with human vision on a difficult vision task
Source: PLoS Comput Biol. 2025 Dec 9;21(12):e1012968. doi: 10.1371/journal.pcbi.1012968 (PMC12707649; doi:10.1371/journal.pcbi.1012968)
Supplement: S7 Text — (PDF) [file pcbi.1012968.s007.pdf]

**S7 Text. Evaluation of regularised Resnet 18 models that match human accuracy** We train Resnet 18 models with dropout after each CNN block with 0.9 probability to drop the output and with subset of training data i.e 2000 for MNIST and 6000 for Fashion MNIST. The dataset was chosen to match the accuracy of the model to human accuracy for the level 11 difficulty. Finally we train models for all other difficulty levels using same training data. Then all models are evaluated on test sets of every difficulty level. The results are presented in Fig 1. We observe that even with high level of regularisation, the Renet 18 models are overfit to the difficulty levels of their training. They perform the best when evaluated on difficulty levels close to their own difficult level. Specifically for extreme case of level 17 models which are trained on a much difficult set, it is unable to perform well on the easier level 9 test set.

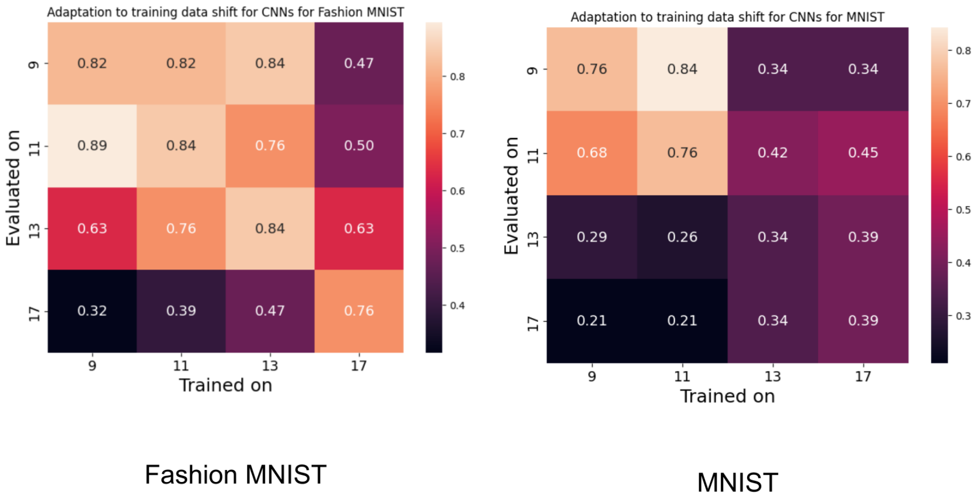


**Fig 1. Regularised Resnet 18: Performance under distribution shift** Evaulation of performance of Regularised Resent 18 models trained and evaluated for different difficulty levels for Fashion MNIST (left) and MNIST (right) dataset respectively. Observe how the models perform the best on and around the difficulty levels that they are originally trained on. Specially models trained on difficulty level 17 perform worse on a much easier difficulty level of 9.
