# Supplementary material for: Comparing a computational model of visual problem solving with human vision on a difficult vision task
Source: PLoS Comput Biol. 2025 Dec 9;21(12):e1012968. doi: 10.1371/journal.pcbi.1012968 (PMC12707649; doi:10.1371/journal.pcbi.1012968)
Supplement: Attachment — Submitted filename: Response to Reviewers.pdf [file pcbi.1012968.s008.pdf]

We would like to thank the reviewers for examining our work. We feel that our revised manuscript has greatly benefited from the thorough and helpful comments. Thanks to the comments of the reviewers, we have now considerably changed the manuscript with additional analysis and information to improve the content of the manuscript and its readability. In the following, we will respond to the reviewers' comments point by point

Reviewer #1:

I found the motivation of this line of research difficult to follow. The task is so difficult it is difficult to know how it relates to the challenges the human visual system confronts. The authors attempt to link the task to more familiar problems such as when the write:

“This approach allows us to use static images for a dynamic and complex image processing task involving iterative steps, tackling a computational problem similar to visual inference and control in a dynamic setting such as for self-driving cars, where each inference step depends on the prior context.”.

But it is hard to see the relation of this task to the process of visual processing and processing of dynamic images. If humans could easily identify these images, then the logic of studying these images would make more sense to me – then DNNs would also need to support these visual functions if they are to be models of human vision. But the importance of matching quite poor performance is less clear to me. The authors need to do a better job motivating the phenomenon they are studying.

**Response: Thank you very much for taking the time to read our manuscript and thank you for this insight. We have now added more context in the manuscript's introduction to clarify the need for this task. In particular, we have now changed the quoted sentence to better reflect the key aims of the paper: “This approach allows us to use static images for a dynamic and complex image processing task involving iterative steps that mimic problem-solving tasks in other domains. For instance, trying to identify the approaching person in a dark alley is akin to a problem-solving task, as it requires combining low-quality bottom-up cues with prior knowledge and iterating over many possibilities.”**

**In this context, the poor performance arises from the difficulty of the task, which is part of the experimental setup. Our goal is to study visual problem solving, and thus, the solution rates necessarily have to be somewhat low (otherwise there is no problem to solve). Humans can still solve the task fairly above random accuracy, i.e., above 60% of the images in a 10-class classification. This, along with the human attempts to find the solutions and draw multiple image hypotheses in the process, shows that this level of difficulty to solve these images is well above random but still is difficult enough that many images require a bit of iterative reasoning over the images to arrive at or at least confirm the solution, making this visual task more similar to a problem-solving task.**

It is also the case the proposed solution of the model seems entirely unrelated two what how humans like reason under these conditions (trying to find lines in the noise). The author write:

“The constellations image is solved by generating candidate solutions with a GAN and refined using a genetic search conditioned on best fitting of the solution outlines to the dots on the constellation image”.

Is the claim that that humans also use GANs and genetic search? If not, what is the claim? The

analyses of the accuracy levels, errors and confusion, does not seem (at least to me), sufficient to lend any support to the claim that human visual system is solving the tasks using similar algorithms.

**Response: Thank you for pointing this out. The claim is not exactly the use of GANs and genetic search in humans, but the use of generative models of the world along with the use of search for reasoning over the candidate solutions with those generative models. The type of generative model and the nature of search are still a matter of further research and an active question. In this work we show the merits of search using a multi-hypothesis heuristic search (evolutionary search) as compared to greedy (gradient descent search) in high level latent space of a generator (GAN).**

**We thus added to the final part of the manuscript a clarification of the claim: "It is important to note that we are not claiming that the human brain necessarily implements genetic algorithms, rather the aim of this work is to explore whether a generative search approach can approximate how humans approach complex visual problem-solving. We show that GenSearch captures some dynamics that are observed in human behaviour, such as iterative hypothesis refinement, diversity of solutions, and occasional sudden shifts. These parallels suggest that search over generative representations may be a useful framework for understanding aspects of human visual inference in visually ambiguous contexts."**

**We now also discuss (in section discussing the limitations of Gensearch) how the search process in humans is more dynamic than GenSearch as humans can traverse between levels of feature hierarchies during the process i.e. we can search for and hence do conditional generation based on many levels of features like lines, curves, parts of object like a arm or wing and not only the high level candidate search like the one we do with GAN.**

The authors also claim that their model does a better job than a ResNet18 model given that performance was too high when trained on these images (or too low for Clip on 0-shot testing). But ResNet18 could have been given less training to get training set to human-like levels. Would error patterns be similar under these conditions? In any case, not suggesting this be done, but not sure the error patterns the author relying on provide strong support for their algorithm.

**Response: The higher accuracy of the Resnet models is used in synergy with its GradCam and change of train test distribution analysis to show that probably the algorithm it's using after being trained on the constellations dataset is overfit to classifying these images and is not the same algorithm that humans are using (using some candidate image generation and search). The higher accuracy is only one of the arguments that work with the other major arguments about the training using 60,000 images, overfitting to the difficulty level so much that it performs much worse on a lower difficulty level when it's more different from the one it was trained on, see Fig. 8a and 8b.**

<u>Reviewer #2:</u>

I did enjoy reading the manuscript. I think it can be improved in many ways -

**Response: Thank you for reading and enjoying our manuscript.**

1. The description of the algorithm in lines 85-103 is super useful but needs more clarification.
a. "The initial population is randomly sampled..". Population of what exactly?
b. Needs a step that generates images with the GAN
c. Steps 2 and 3 should perhaps be combined
d. Confusing usage of "point mutation and crossover" without explaining what it means first.

**Response: Thank you for highlighting these specific issues. We have now updated the text to improve the clarity of these sections.**

2. Can the correlation between confusion matrices be calculated for humans and resnet18? Reaction time could also be predicted with a recurrent neural network (Goetschalckx et al. 2023)

**Response: Yes, we have calculated the confusion matrices between humans and ResNet 18, the correlations are slightly lower than GenSearch at 0.68 and 0.66 for Fashion MNIST and MNIST. The figures have been added to the relevant section of the text for comparison. The confusion matrices are added to the supplementary material 'S3 text'. With very high accuracy of above 90% for both models, the correlation captures the matching correct solution classes, but there are not many mistakes made by these models to reflect the similarity to the mistakes made by humans.**

**We havent focused on reaction times specifically because in our experiments with humans, the participants were not prompted to immediately submit their initial guesses when they recoganise the image, instead they were prompted to the draw the solution as part of the iterative solving process, which could contribute to variation in capturing reaction time as compared to when they actually identified the object. This could specifically skew the reaction times for stimuli that are identified immediately upon presentation, as the total time is then dependent on the drawing times by the participants, which tended to vary based on how much detail was added.**

<u>Reviewer #3:</u>

Khajuria, Tulver and Aru propose a way to formalize the idea of visual perception as an "analysis-by-synthesis" process, in which a generative model is used to propose solutions to a perceptual problem, which are then refined based on their compatibility with visual input. Here, this process is implemented as an evolutionary search over latent vectors in a pre-trained generative model (a GAN). The task is to interpret "constellation" images, in which an object's outline is shown as a collection of dots amidst randomly positioned nuisance dots, as objects belonging to one of a set of categories. Specifically, images from the popular computer vision datasets MNIST and FashionMNIST are used to generate the stimuli. Vectors in the GAN's latent space are evaluated by their compatibility with the dot arrangement shown in a given image. This provides a fitness function by which the most promising vectors are selected, which then undergo crossover and mutation. This process is iterated for 30 generations, yielding vectors that are well-adapted to the image. Classifying the underlying object category from the final solution images yields an accuracy that is similar to that of human subjects (~60% for both MNIST and FashionMNIST). The classification errors made by this model are also similar to those of humans. Moreover, the object shapes inferred by the model were also similar to those drawn by human subjects. The model is compared with a set of baseline deep learning models, finding that these models either failed at the task, or exhibited unrealistically high accuracy.

I found the approach described in this paper quite original and interesting, but I also feel that there are a few issues that the authors should address.

**Response: Thank you! We hope we have now addressed these issues in our revision.**

My main question is a conceptual one: what are the claims precisely, with respect to the search process? The authors have chosen to implement the search over latent parameters as an

evolutionary algorithm. This is a very interesting choice, as search algorithms such as evolutionary search are more commonly used for discrete tasks with a combinatorial structure, such as planning or program synthesis. This choice has several implications. Particularly, the main implication is the ability to maintain a “library” of diverse solutions to the search problem. This is in contrast, for example, to gradient descent, which would more greedily explore a specific direction in the search space. The authors hint at the possibility that a more open-ended evolutionary search might be a better model of perceptual analysis-by-synthesis in humans, for example by mentioning multi-stable phenomena in vision, but this claim is not made explicit nor explicitly tested. In fact, I believe that the specific choice of an evolutionary algorithm is the main novelty of this work, as the idea of analysis-by-synthesis per se has been explored in depth already, but to my knowledge never implemented in this particular way.

**Response: The main claim is that search algorithms like evolutionary algorithms with their ability to maintain and stochastically operate on multiple candidate hypotheses, make them a likely candidate for the search process used in many processes of human cognition like perception and reasoning. We have now added to the final part of the manuscript a clarification of the claim: “It is important to note that we are not claiming that the human brain necessarily implements genetic algorithms, rather the aim of this work is to explore whether a generative search approach can approximate how humans approach complex visual problem-solving. We show that GenSearch captures some dynamics that are observed in human behaviour, such as iterative hypothesis refinement, diversity of solutions, and occasional sudden shifts. These parallels suggest that search over generative representations may be a useful framework for understanding aspects of human visual inference in visually ambiguous contexts.”**

**We chose to implement the search as an evolutionary algorithm rather than optimising a single gradient-based objective because the task lacks a straightforward differentiable objective function. The problem was formulated as finding the contour in GAN that maximises the number of dots it passes through in the constellation image. However, calculating this is a discrete matching problem, making the loss non-differentiable. Hence, we could not use gradient descent over this problem. Evolutionary search, on the other hand, is well suited in this context due to its ability to optimise over a discrete fitness function, making it not only a theoretically plausible candidate but also a computationally appropriate solution for this type of visual problem.**

**We have now made changes to the discussion section to clarify the claims and make them more explicit. Further, we formulated and implemented a gradient-based search variation for GenSearch for comparison and included the results in a separate section (Results-> comparison with gradient descent search).**

So I think an explicit comparison with a different search method would substantially strengthen the paper. The main alternative hypothesis, in my opinion, would be a search for latent parameter configurations via gradient descent, for two reasons: 1) Test-time optimization via gradient descent has been extensively used in energy-based models, showing that it is a viable method to solve many perceptual and reasoning tasks (Mordatch 2018, for example, used gradient descent to arrange dots into configurations that matched a particular goal concept, which might bear some resemblance to the task of finding objects in noisy dot configurations used here). 2) While there are indeed, as the authors point out, phenomena in human perception that suggest multiple explanations of a perceptually ambiguous stimulus are being considered, such as multi-stable stimuli, most computational models of perceptual ambiguity resolution (including object recognition in clutter) through recurrence in neural networks formalize the task as one of evidence accumulation in favor of a specific hypothesis (e.g. Thorat, Aldegheri & Kietzmann 2021, Goetschalckx, Govindajaran et al. 2023). While subtle differences in task design might lead to different search algorithms being more effective, this shows that the alternative hypothesis of a greedy search in one promising direction,

whether implemented through gradient descent or recurrent dynamics, is worth testing. The comparison between evolutionary and gradient descent-based search could be implemented by making the pipeline from latent noise vector to contours fully differentiable. The GAN's generator is already a neural network, and the edge detection could be replaced by a convolutional layer with fixed Gabor-like kernels. Both of these could be frozen, and gradient descent on the latent vector itself could be used to find optimal solutions. Of course, the authors should let me know in case there are practical difficulties with this approach which I am missing.

**Response: As mentioned in the previous comment, the initial problem in implementing a gradient-based solution to this problem is the lack of a straightforward differentiable objective to optimise such a solution. Currently, the objective function or the fitness function is the outline passing through the maximum number of dots in the image. But this by itself is a non-differentiable matching problem, especially if you want to consider solutions with a certain radius of tolerance.**

**However, considering your suggestion, we have now formulated and implemented an approximate loss to mimic this loss using a heatmap at points of a particular constellation image and matching the generated solution edgemap to this heatmap masked at the points of activation in the heatmap. We tried three measures of similarity, i.e., MSE, cross-entropy and dot product similarity between the two image maps. Finally, the results are reported in the manuscript with dot product similarity, as it worked better than others to optimise for the best number of dots touched by the solution's edges. The gradient-based method seems to converge to a more local solution that touches the same or even more number of points as the evolutionary search (evaluated using the same function we used for evolution-based GenSearch), but finds a local solution with artefacts and spurious shapes that overlap with the dots. This preliminary finding suggests a regularising effect of the evolutionary search due to maintaining multiple solutions and the stochastic operations on those solutions. We have discussed these findings both in a new section (Results-> comparison with gradient descent search) in the manuscript as well as in the discussion section of the paper.**

A more methodological issue regards the analysis measuring the overlap between GenSearch and human drawings. It is hard to make sense of an absolute IOU quantity, without a baseline to compare it with. The most straightforward baseline would be to add the IOU between human drawings and the ground-truth contours, to show whether GenSearch overlaps with human drawings merely because they are both trying to get at the same answer (IOU(humans, groundtruth) ≥ IOU(humans, gensearch)), or if it also makes similar mistakes (IOU(humans, groundtruth) < IOU(humans, gensearch)). Additionally, to clarify the same question, the correlation between IOU on individual images for GenSearch and humans should be reported, to show whether humans and GenSearch tend to perform worse or better on the same images (or categories, but individual images might be a more fine-grained and thus sensitive analysis).

**Response: IOU(humans, groundtruth) 0.69 is greater than IOU(humans, gensearch) 0.6 for MNIST and IOU(humans, groundtruth) and for Fashion MNIST IOU(humans, groundtruth) 0.76 is greater than IOU(humans, gensearch) 0.52. It does suggest that it can't be concluded using IOUdots numbers that the overlap corresponds to an overlap of mistakes. But the metric is primarily used to compare against other methods, such as pix2pix. To quantify specifically the overlap on mistakes we now additionally measure IOU only on the mistake points (removing ground truth points for both search and human drawings) and report it alongside the original IOU readings which are 0.43 for MNIST and 0.58 for Fashion MNIST, with some of the those individual IOUs being very high, closer to 0.9 on a few individual images.**

I find the comparison with pix2pix particularly interesting and informative, since it looks like a perfectly suited model for the task which is based on completely different principles from the GenSearch model.

However, it is hard to interpret its low performance. Are the authors confident that it is due to an intrinsic incompatibility between the model and this task, and not e.g. to a poor choice of hyperparameters? Within the limitations of the computational power at their disposal, the authors should try to explore hyperparameters to make pix2pix as competitive as possible: right now, its performance is very poor, as shown in Figure 8, making it hard to meaningfully compare it with humans. Of course, it is possible that the model is intrinsically unsuited for this task, so I'm not requiring the authors to reach a given level of accuracy. But at least, showing that its poor performance is consistent across hyperparameters would make the claims about the superiority of GenSearch in approximating human behavior more convincing.

**Response: We have already trained the pix2pix network by optimising the generator architecture and using a suitable training schedule recommended to train such GANs. The only hyperparameter that we found has the most impact and is now reported in supplementary material S4 text is the starting learning rate. With a more systematic search, we were able to find a bit better setting to train the model, but the IOU dots for the models still stay considerably lower than that of GenSearch. Also, we added an additional experiment where we use the model trained on MNIST to solve the Fashion MNIST images, and the performance is considerably close to the dataset-specific models. This makes us infer that Pix2pix does not really use the high-level model of the object categories and relies on low-level cues, such as the distance between the dots, to connect the dots. The distance between the dots in the image is uniform for the object dots and varied for the noise dots. This local information may be used by the network to solve the images, as many solutions have connected dots that should not form the contour according to the image, but are close enough to the distance between the contour dots.**

Beyond these major points, below I list some minor and more specific issues that the authors should address:

- In Figure 2b, the bar plot does not include error bars. Adding them would be good scientific practice, at least for human subjects data.

**We have now added error bars for both human accuracy and GenSearch (3 random seeds).**

- In Figure 4, which shows human mistakes next to GenSearch mistakes, it would be useful to see the ground-truth contours as well, to make it clearer what exactly the mistake was. Also, for this and other figures, including a few more examples would be good (unless the journal formatting guidelines make that difficult).

**Thank you, we have now added the original contour and added a few more examples in this figure.**

- In general, I find the human experiment sample sizes to be on the low side, but in particular, I do not think that having only two participants perform the task at the difficult level is sufficient. Unless there are major practical impediments in doing so, the authors should collect a larger sample on this difficulty level, and if possible on other difficulty levels (e.g. 9, 13) such that the results in Figure 7a can be more properly assessed, with confidence intervals as well.

**We have now added 4 more participants to both datasets for difficulty level 17. While that could be seen as a small sample for certain psychological experiments, there are also many domains (e.g., psychophysics) that rely on even smaller samples to display robust perceptual effects. The overall aim of doing the experiments for this difficulty level was to show how, at such a high difficulty, humans find it extremely challenging to identify any shapes. The results overall, with the mean accuracy of 0.22 (+-0.12) for Fashion MNIST and 0.18 (+-0.03) for MNIST, are fairly robust in illustrating that these images are very difficult to solve for humans. Many participants in our new batch also expressed frustration and reported that solving this level**

**was really hard and stressful, so we concluded this study with 6 participants in each dataset.**
- Somewhere either in the main text or supplementary materials, examples of the full color images generated by the GAN should be added, to get a clearer idea of the workings of the model. Following the analogy implied by this model, the GAN outputs would be something like the mental images of the object evoked by the constellation stimuli, so seeing these images would be an interesting qualitative measure of how humanlike the model is.

**We have now added images generated by the GAN in supplementary material S5 Text, Images. The images for MNIST and Fashion MNIST are themselves greyscale, hence the generator also generates only greyscale images.**
- At line 357, p. 15, there is a typo: the table number is missing at the very end of the line.

**Thank you for pointing out this mistake.**
