# Supplementary material for: Comparing a computational model of visual problem solving with human vision on a difficult vision task
Source: PLoS Comput Biol. 2025 Dec 9;21(12):e1012968. doi: 10.1371/journal.pcbi.1012968 (PMC12707649; doi:10.1371/journal.pcbi.1012968)
Supplement: Attachment — Submitted filename: Response_to_reviewers.pdf [file pcbi.1012968.s009.pdf]

Reviewer #1: I think the authors for responding to my questions, I understand the motivation of the project better now. But I'm still not convinced that that the findings merit publishing in PLOS Computational Biology. I'm not confident in my assessment, but let me outline my outstanding concerns.

The authors have clarified how the constellation task relates to visual problem solving that the visual system confronts – it is thought analogous to "trying to identify the approaching person in a dark alley... as it requires combining low-quality bottom-up cues with prior knowledge and iterating over many possibilities"

This makes more sense to me now, but I'm not convinced the authors have provided a strong enough test of the correspondence of their algorithm. The model can match overall performance, shows a similar reduction in performance with increased difficultly, and shows a similar pattern of errors (such that similar shapes can be confused). This is contrasted with a ResNet18 that performs near ceiling across levels of noise, which is taken to reflect overfitting.

But presumably it would be quite easy to reduce overall performance of the ResNet18 models, such as providing it less training, and attempt to reduce overfitting by adding some regularisation terms, or dropout, for example. Whether the advantages would still be obtained when more of an effort was testing alternative approaches is not clear to me.

Response: We thank the reviewer for re-assessing our manuscript and for the constructive feedback. We have performed some of the suggested changes to the ResNet training by adding regularisers like dropout and using only part of the dataset to train the models, i.e, 6000 samples for Fashion MNIST and 2000 for MNIST. We find that these relatively underfit models still performed best at their own respective training difficulty level or the levels near its difficulty. As depicted on the figures below, this was especially apparent with the models trained on extreme cases - that is, the models trained on the highest difficulty level 17) performed worse on the easiest level 9, consistent with the idea that the training did not generalize optimally across difficulty levels. We have reported the experiments and results also in Appendix (S7_text).

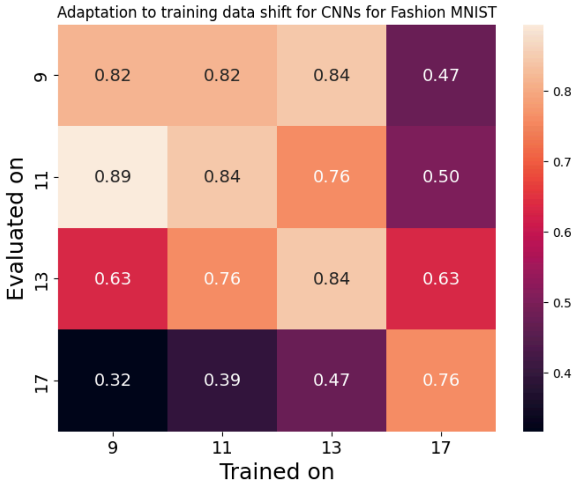

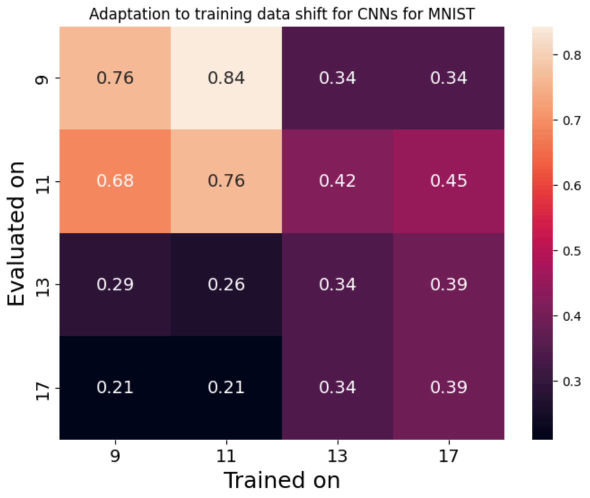

The authors also clarify their theoretical claim, writing: “It is important to note that we are not claiming that the human brain necessarily implements genetic algorithms, rather the aim of this work is to explore whether a generative search approach can approximate how humans approach complex visual problem-solving.”.

So the claim is much more general than this particular algorithm they report, or even evolutionary approach, and I wonder whether this suggests that other architectures would succeed, such as recurrent networks. Indeed, it has been shown that recurrent networks are better able to solve more difficult visual problems, and that these models can converge to solutions rather than a fast single bottom-up pass (Nayebi et al., 2022). Clearly, humans are doing in problem solving context the authors are studying – making multiple attempts to solve the task. So, it feels like the authors are comparing their approach to a bit of a strawman model (ResNet), and that other theoretically motivated approaches might succeed to the same extent.

Response: Thank you for raising this issue. We have now added this clarification to the discussion as a limitation and direction for future work: Furthermore, in our analysis, we compared our model against either feedforward networks or gradient-based search. It will be useful for future studies to explicitly test the behaviour of recurrent neural networks, which have been shown to better capture human behaviour in terms of reaction times and performance trade-off (Spoerer et al., 2020) and outperform their feedforward counterparts under challenging conditions (Spoerer et al., 2017).

Nayebi, A., Sagastuy-Brena, J., Bear, D. M., Kar, K., Kubilius, J., Ganguli, S., ... & Yamins, D. L. (2022). Recurrent connections in the primate ventral visual stream mediate a trade-off between task performance and network size during core object recognition. Neural Computation, 34(8), 1652-1675.

Reviewer #2: Thank you for addressing my comments.

Reviewer #3: I thank the authors for taking the time to address my concerns thoroughly. I do think the paper has been substantially improved. I still have concerns, however, about the way the (updated) results are presented. I think in its current form the manuscript lacks clarity. My concerns regard two results in particular: the comparison with gradient descent, and the IOU results.

Response: Thank you very much for your help with the revision.

- Comparison with gradient descent: the authors have done an excellent job in making the pipeline differentiable and comparing the evolutionary search results with those obtained using gradient descent. I think, however, that these results should be made clearer in the following ways:
1. As far as I can tell, right now only a single example of the gradient-based search results is shown in Fig. 7. Including a few more examples would help the reader to gauge systematic differences between this and the evolutionary search.

Response: We have added a few more examples of the evolution of solutions by gradient descent in the manuscript and additionally in Fig 7.

2. In Table 1, only the classification accuracy for the evolutionary vs. gradient-based version of

GenSearch are reported. In the text, the authors also mention that the gradient-based search obtained a higher overlap score with the dots, but this score is not reported. It should be added to the table, next to the classification accuracy, to highlight the tradeoff.

Response: We have now added the average count of dots per solution to the table comparing gradient-based search to GenSearch.

3. In reporting the results of the gradient-based model, the authors write (p. 10, lines 210-212): “We observe that the gradient-based search finds solutions optimal to the desired objective, i.e., the search increases the number of dots passing through the candidate solution least as high as the top solution obtained using evolutionary search.” This sentence is not grammatical, so I would invite the authors to rephrase it in a clearer way.

Response: In rewriting this section, we have removed this sentence altogether.

4. The authors make claims about the ability of evolutionary search to maintain multiple solutions being key to its success in approximating human performance. They also report the best-performing hyper-parameters of the genetic algorithm in Table 3. It might be useful to clarify to what extent these hyper-parameters are consistent with the hypothesis that the ability to maintain multiple hypotheses is central to the success of GenSearch (e.g. larger mutation rate, larger solution pool or population size…). On p. 15, lines 314-315, the authors write “A large parent selection allows multiple candidate solutions to participate in the next generation, promoting diversity in the search space”, which gets close to the idea that this selection of hyper-parameters works well because of its ability to maintain diversity, but this is not tested explicitly.

Response: Thank you for this suggestion! We conducted an additional study to compare GenSeach in a setup where we restrict both the size of the population and the parents pool by a factor of 10 while increasing the maximum number of generations by a factor of 10. We then performed a hyperparameter search for the best crossover and mutation rates for this setup. We found that the best resulting setup had a lower accuracy (0.44) than the original setup (0.66) on the MNIST dataset. The decrease in performance in this setting is expected, as evolutionary algorithms with smaller populations have the tendency to exhibit lower genetic diversity and can converge prematurely to a more local solution (Harik et al., 1999, Xu and Gao, 1997). Thus, the population size in that manner represents the exploration vs compute trade-off in genetic search. We have added this analysis to appendix S6_Text.

The claim in the paper is a much more general one - we suggest that evolutionary algorithms, unlike other single candidate searches, rely on having multiple candidate solutions by design, and that certain aspects of human problem solving may similarly benefit from sustaining and revising multiple hypotheses during the search process. In the discussion section of the article, we also note that humans likely employ a more flexible and efficient search strategy within the multiple hypotheses compared to our implementation of an evolutionary algorithm, as they are able to dynamically shift between different levels of feature abstraction and use goal-directed cues to guide the search process. This flexibility may allow humans to achieve both diverse exploration and rapid convergence without the need for extensive computational resources.

5. In general, I feel like there are many claims about similarities between GenSearch and human cognition which are not substantiated by the data. One example is the aforementioned ability to maintain multiple solutions: the only evidence for this being related to the search process in humans are the two examples in Figure 5. While these look compelling, two individual examples are not sufficient to draw broad conclusions. Moreover, other tests of this similarity in search dynamics, namely the correlation between convergence and reaction times, yield inconclusive results. I would thus encourage the authors to either (A) conduct more thorough tests of the relation between GenSearch and the search for the best-fitting solutions by human subjects, or (B) express any claims about similarities, or mechanistic explanations for GenSearch's superior performance to other methods, in more tentative terms. For example, on page 15, lines 322-323, they write that GenSearch's superior classification performance to gradient descent "showed the regularising benefit of the multi-hypothesis stochastic nature of the evolutionary search". As there are several differences between evolutionary search and gradient descent beyond the ability to hold multiple hypotheses, I would rephrase this as "suggests" rather than "showed". Similarly, on p. 16, lines 337-339 "GenSearch, by maintaining multiple hypotheses, provides a natural heuristic similar to that of humans considering different hypotheses in search of a visual solution". While I think it is fine to speculate that considering different hypotheses is what makes GenSearch's performance superior to gradient descent (in fact I suggested using gradient descent as a baseline for this reason!), I don't think the present data justify such strong claims.

Response: Thank you for pointing this out. We have changed the wording on our claims to be more tentative. As you rightly pointed out, the nature of this work is indeed exploratory, as we aim to illustrate how a search process that maintains multiple hypotheses has certain advantages over traditionally accepted models of vision, both in terms of performance and in capturing aspects that may be consistent with aspects of human problem-solving. We have also made it more clear in our discussion that we don't really believe that this is the exact algorithm that humans use, especially due to its high computational demands and limited scalability to the complexity of real world objects and search spaces.

- IOU results: in their response, the authors do mention the IOU between ground-truth and human drawings. I believe that this would be informative to include in the table (or turn the table into a figure), as an upper bound. Also, it is informative to show that the overlap between human drawings and ground-truth is still higher, showing that humans are still better than the model at capturing the shape of the 'hidden' objects. Perhaps, but this is just a suggestion, it could even be interesting to show this at different levels of difficulty, since with more noisy and ambiguous stimuli human subjects are likely to rely on their priors more, and GenSearch might be able to capture these priors. Concerning the overlap of mistakes, I think this should also be reported in the figure/table together with the other IOU results, to get a clear and complete picture. Even more importantly, the overlap in mistakes between pix2pix and humans should also be included, to get an apples-to-apples comparison with GenSearch.

Response: We have now added the IOU ground-truth between humans and drawings to the table caption as a baseline. Also, the number of IOU mistakes for humans, Gensearch and pix2pix have been added in a single table in Table 2.

In general, I stand by my initial assessment that this is a valid and interesting work. I do think, however, that it is more of a proof-of-concept than a conclusive finding. With the modifications I recommended,

including a change in wording to highlight the speculative nature of some conclusions, I believe it will be ready for publication

References:

Harik G, Cantú-Paz E, Goldberg DE, Miller BL. The gambler's ruin problem, genetic algorithms, and the sizing of populations. Evolutionary computation. 1999 Sep;7(3):231-53.

Xu Z, Gao Y. Characteristic analysis and prevention on premature convergence in genetic algorithms. Science in China Series E: Technological Sciences. 1997 Apr;40(2):113-25.

Spoerer CJ, Kietzmann TC, Mehrer J, Charest I, Kriegeskorte N. Recurrent neural networks can explain flexible trading of speed and accuracy in biological vision. PLoS computational biology. 2020 Oct 2;16(10):e1008215.

Spoerer CJ, McClure P, Kriegeskorte N. Recurrent convolutional neural networks: a better model of biological object recognition. Frontiers in psychology. 2017 Sep 12;8:1551.
